# Bispecific antibody engineered from two non-protective antibodies confers full protection against anthrax toxin through inhibition of PA83 cleavage

**DOI:** 10.1128/spectrum.00588-25

**Published:** 2025-08-29

**Authors:** Zhengshan Chen, Guanying Zhang, Jin Han, Zheng Zhu, Meirong Wang, Ting Fang, Zeya Li, Yunzhu Dong, Jianmin Li, Changming Yu, Xiangyang Chi

**Affiliations:** 1 Laboratory of Advanced Biotechnology, Beijing Institute of Biotechnology, Beijing, China; LSU Health Shreveport, Shreveport, Louisiana, USA

**Keywords:** *Bacillus anthracis*, protective antigen, non-protective antibody, bispecific antibody, neutralizing mechanism

## Abstract

Anthrax is a highly contagious zoonotic disease, posing a great threat to human life and health. Protective antibodies against anthrax protective antigens (PA) can play a crucial role in the prevention and treatment of anthrax infections. However, the functions and mechanisms of the large number of non-protective antibodies elicited during the infection or vaccination of anthrax have not been clearly understood. Here, we found that two non-protective antibodies, 2A6 and 8A7, exerted an enhanced neutralizing effect both *in vitro* and *in vivo*. In the monoclonal antibodies (mAbs) combination, 8A7 increased the aggregation of PA, which might favor the inhibition of PA pore formation by 2A6. Structural analysis demonstrated distinct interaction patterns between the monoclonal antibodies, with 8A7 primarily interacting with domain 3 of the PA protein, while 2A6 establishes direct contact with domain 2. We further constructed bispecific antibodies in the form of DVD-Ig. The 2A6-8A7 conserved the parental antibody functions in promoting PA aggregation and inhibiting pore formation and simultaneously exhibited a distinct neutralizing mechanism through preventing PA83 cleavage. This study provides new insights into how the non-protective antibodies work in collaboration *in vivo* and opens new avenues for optimizing future anthrax prevention and control strategies.

**IMPORTANCE:**

Anthrax remains a bioterrorism threat and public health challenge, where protective antibodies targeting protective antigen (PA) are critical for treatment. However, the role of non-protective antibodies—abundant yet poorly understood components of immune responses—has been unclear. This study reveals that two non-protective antibodies (2A6 and 8A7) unexpectedly block anthrax toxin activity in combination. By combining these antibodies, we observed enhanced PA aggregation and inhibition of toxin pore formation, with structural results showing their distinct binding sites on PA. Furthermore, engineered bispecific antibodies derived from this pair not only exhibited superior neutralization but also uncovered a novel mechanism: blocking the cleavage of PA, a critical step in toxin activation. These findings challenge the traditional view of non-protective antibodies as biologically inert, offering a transformative strategy to repurpose them into potent therapies. This work advances anthrax treatment design and deepens our understanding of how antibody combinations mimic natural immunity to combat pathogens.

## INTRODUCTION

*Bacillus anthracis*, a Gram-positive and spore-forming bacterium, is the causative agent of anthrax, which mostly affects grazing livestock but also poses a threat to human health. The main pathogenic factor of *Bacillus anthracis* is the tripartite toxin, which includes three proteins: protective antigen (PA), lethal factor (LF), and edema factor (EF). Full-length PA is an 83 kDa protein that binds to target cells and, through enzymatic cleavage, transforms into the active form PA63, which can spontaneously polymerize into heptameric oligomers (1). Upon PA oligomers binding to LF or EF molecules, the complex enters the cell through endocytosis. As the environmental pH decreases, PA63 oligomer undergoes a conformational change to a heat and SDS-stable form and builds a central pore that allows the translocation of LF or EF into the cytosol (2). LF is a zinc-dependent protease specific to the mitogen-activated protein kinase kinase (MKK/MAPKK) family. It assembles with PA into lethal toxin (LeTx) and cleaves MKK to initiate the cell apoptosis program (3). EF is a calmodulin-dependent adenylate cyclase that can potently elevate intracellular cAMP levels and cause edema (4).

Evidently, PA plays a pivotal role in the damage to host cells during anthrax infection. PA contains four folding domains (5). Proteolytic activation by furin occurs at the cleavage site within Domain 1 (residues 1–258), generating a nicked form of PA83 as the precursor of the heptameric PA63. Domain 2 (residues 259–487) forms a transmembrane pore to serve as the portal of entry of EF and LF into the cytosol. Domain 3 (residues 488–595) is believed to mediate self-association of PA63 (6). Domain 4 (residues 596–735) is primarily involved in binding to cellular receptors, and Domain 2 has also been shown to participate in this process (7). Two related cell surface receptors that bind PA83 have been identified: ANTXR1 (tumor endothelial marker-8) and ANTXR2 (capillary morphogenesis protein 2).

Neutralizing antibodies targeting PA have been shown to protect animals from anthrax toxin challenges through inhibiting toxin-cell interactions by blocking the binding of PA83 to cellular receptors (2, 8), inhibiting enzymatic hydrolysis of PA83 (9, 10), preventing the polymerization of PA63 into heptamers, or impeding the conformational transition from the pre-pore to pore state (11, 12). The mixed application of monoclonal antibodies (mAbs) can elicit enhanced and augmenting effects. Various studies have demonstrated that the potency of protective mAbs can be augmented additively or synergistically by adding other protective mAbs (13–16) or non-protective antibodies (17–20). However, the functions and mechanisms of the combination of non-protective antibodies are rarely explored and understood.

Through combining two mAbs targeting different antigens or different epitopes of the same antigen, bispecific antibodies could bind two antigenic sites at the same time and thus confer better therapeutic effects and protective activities and even introduce novel neutralizing mechanisms (21–23). The application of bispecific antibodies derived from two protective antibodies has been shown to be effective in many studies, but the function of bispecific antibodies originating from non-protective mAbs has been little investigated. Here, we demonstrated the enhanced neutralizing effect of two non-protective mAbs, 8A7 and 2A6, explored the neutralizing mechanisms of the mAbs combination, and further constructed and characterized the derived bispecific antibodies in DVD-Ig form. Our results demonstrate that non-protective mAbs originating from combination or bispecific antibodies could confer neutralizing activity both at the cellular level and in animal models, representing new insights into the development of therapeutic antibody drugs.

## RESULTS

### 2A6 and 8A7 confer enhanced neutralizing effect against LeTx

**Fig 1:**
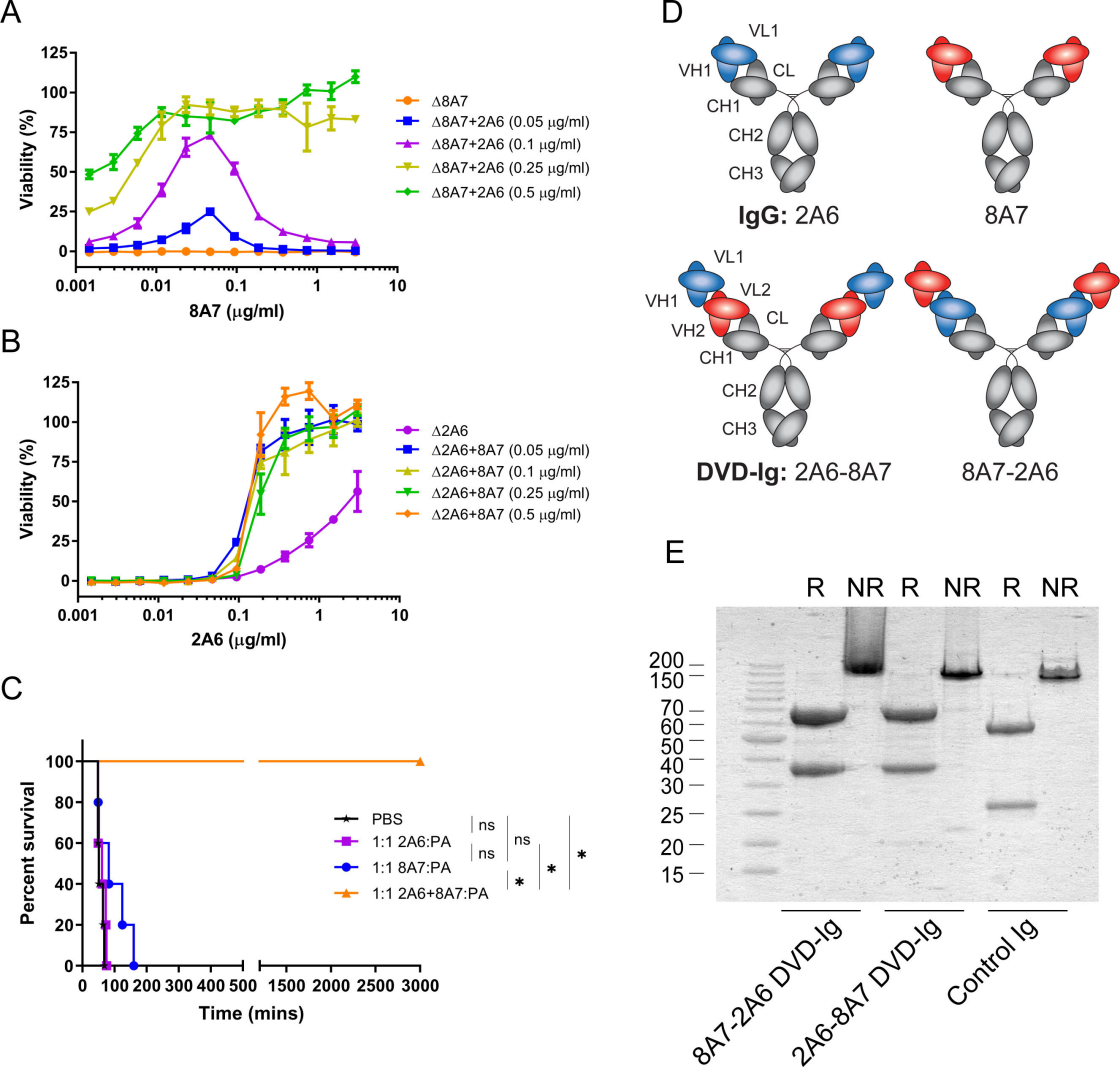
Combination of antibodies and construction of bispecific antibodies. (**A and B**) The neutralizing activity of the combination of antibodies *in vitro* was measured by the TNA assay with J774A.1 cell. (**A**) The concentration of 2A6 was kept constant, and that of 8A7 was titrated. (**B**) The concentration of 8A7 was kept constant, and that of 2A6 was titrated. "Δ" represents the antibodies whose doses are variable. Data represent the mean ± SD calculated from three individual experiments performed in duplicates. (**C**) Percentage survival of Fisher 344 rats challenged with LeTx (10 µg of PA and LF each) and mAbs (2A6 and 8A7 used alone or in combination). Each group contains five animals, and *P* values were calculated by the log-rank (Mantel-Cox) test: *P < 0.05. (**D**) Schematic diagram of the molecular structure of bispecific antibodies and their parental antibodies, with blue representing the V region of 2A6 and red representing the V region of 8A7, respectively. (**E**) SDS-PAGE showing the expression level and molecular weight of the mAbs and the bispecific antibodies, under reducing conditions and non-reducing conditions, respectively.

In our previous study, a series of fully human mAbs targeting PA were screened from volunteers vaccinated with recombinant anthrax PA vaccine. Among these mAbs, two non-protective antibodies, 2A6 and 8A7, showed enhanced neutralizing effect against LeTx when tested in the toxin neutralization assay (TNA) with J774A.1 cells (Fig. 1A and B). When used alone, 8A7 could not neutralize LeTx, while 2A6 showed moderate neutralizing activity at high concentration. In contrast, the combination of 8A7 and 2A6 could achieve 100% neutralization. Interestingly, when the concentration of 2A6 was fixed at 0.05 or 0.1 µg/mL, neutralization activity peaked at a certain concentration for 8A7. When the concentration of 2A6 was fixed at 0.25 or 0.5 µg/mL, neutralization activity elevated as the concentration of 8A7 was increased (Fig. 1A). Differently, the neutralization activity improved similarly as the concentration of 2A6 was increased regardless of the amount of 8A7 (Fig. 1B). The results revealed that the enhanced protection against LeTx required the simultaneous interactions of the two mAbs on PA and that the synergy decreased in an excess of 8A7.

The enhanced neutralizing effect of 2A6 and 8A7 was also observed in rats challenged with LeTx (Fig. 1C). Rats treated with PBS served as the death control, and all died within 100 min after LeTx challenge. Rats treated with 2A6 all died within 100 min, while 8A7 slightly prolonged the survival time of treated rats, yet they all died within 200 min. The combination of 2A6 and 8A7 provided full protection for the challenged rats, and none of them died during the observation.

Based on the enhanced neutralizing effect of 2A6 and 8A7, we sought to explore the function of bispecific antibodies derived from these two mAbs. Therefore, we constructed two bispecific antibodies in DVD-Ig format, named 2A6-8A7 and 8A7-2A6, with the difference lying in which antibody’s variable region was connected to the constant region (Fig. 1D). The two variable regions of the heavy or light chains were connected by natural flexible linkers ASTKGP or TVAAP, respectively. As shown in SDS-PAGE results, the heavy chains of the bispecific antibodies were approximately 65 kDa, and the light chain was approximately 37.5 kDa, while the traditional IgG antibody had a heavy chain of 55 kDa and a light chain of 25 kDa (Fig. 1E).

### DVD-Ig bispecific antibody 2A6-8A7 potently neutralizes LeTx

**Fig 2:**
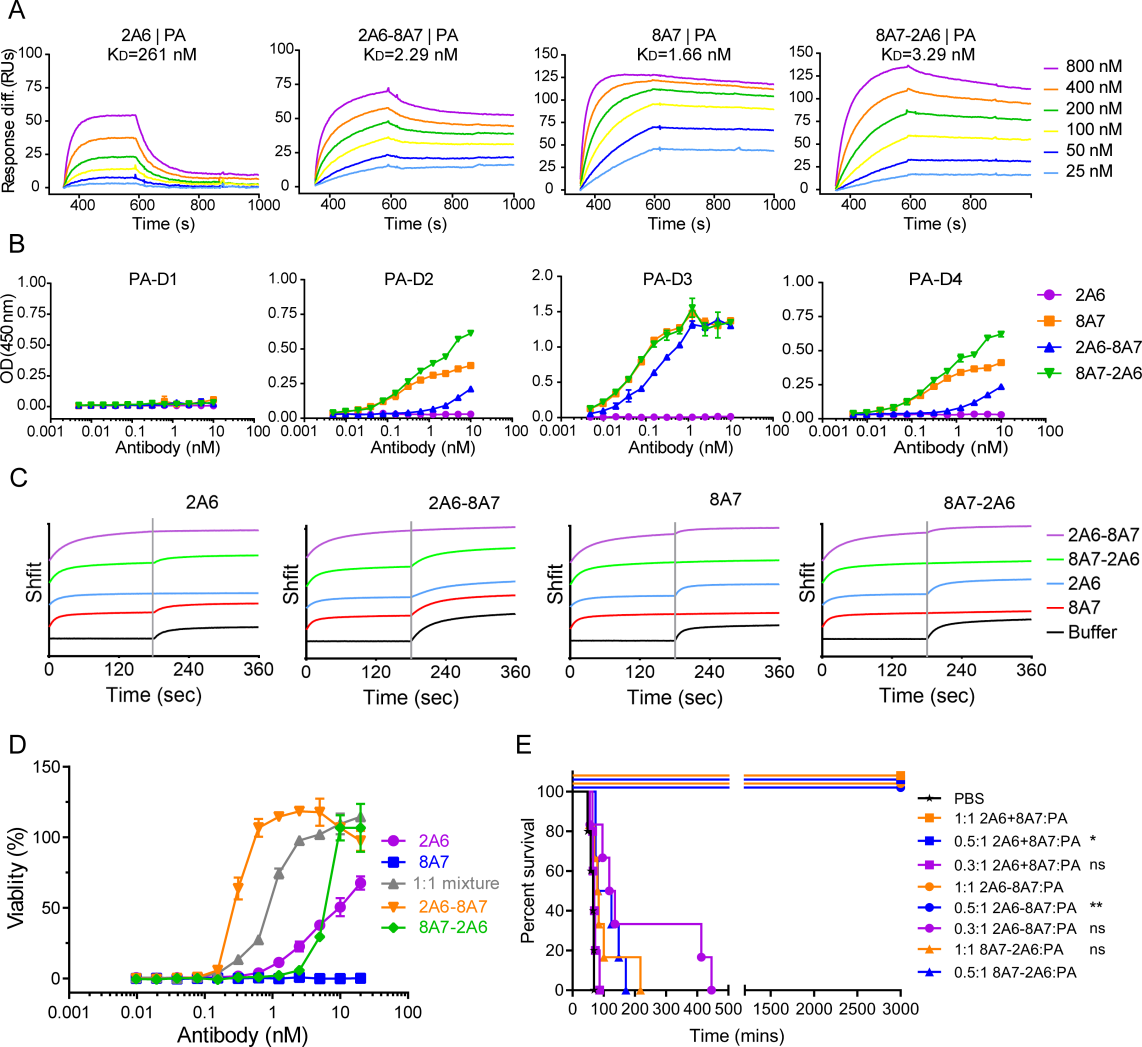
The affinity and domain specificity of bispecific antibodies. (**A**) Analysis of binding kinetics of the interaction between the mAb or the bispecific antibody with PA using the SPR assay. Reported *K*_*D*_ values correspond to avidity due to the utilization of PA constructs. (**B**) Enzyme-linked immunosorbent assay (ELISA) assays showing the binding efficiency of the bispecific antibodies and their parental antibodies to different domains of PA. Data represent the mean ± SD pooled from two individual experiments performed in duplicates. (**C**) The sequential binding patterns of antibodies to PA were revealed by biolayer interferometry (BLI) assays. The legends on the right side of the figure represent the first bound antibodies, and the name on each blot represents the second bound antibody. (**D**) The neutralizing activity of bispecific antibodies *in vitro*. Data represent the mean ± SD pooled from three experiments performed in duplicates. (**E**) Percentage survival of Fisher 344 rats challenged with the mixture of LeTx (10 µg of PA and LF each) and mAbs (the two bispecific antibodies and the combination of their parental antibodies with various molar ratios). Each group contained five animals, and *P* values were analyzed via Log-rank (Mantel-Cox) test compared with the PBS group, *P < 0.05, **P < 0.01.

To assess the function of the bispecific antibodies, surface plasmon resonance (SPR) experiments were conducted to determine the affinity of the bispecific antibodies and their parental antibodies to PA (Fig. 2A). The results revealed that both bispecific antibodies exhibited a strong affinity for PA (*K*_*D*_ = 2.29 nM for 2A6-8A7 and *K*_*D*_ = 3.29 nM for 8A7-2A6), comparable to that of the parental antibody 8A7 (*K*_*D*_ = 1.66 nM). In contrast, 2A6 demonstrated weak affinity to PA with a *K*_*D*_ of 261 nM, mainly due to the rapid dissociation rate.

The binding activity of bispecific antibodies with different domains of PA was further determined using enzyme-linked immunosorbent assay (ELISA) (Fig. 2B). Antibody 2A6 could only bind the entire PA molecule but not any of the four structural domains of PA, and 8A7 showed strong binding activity to domain 3 (EC_50_ = 0.06 nM), moderate binding activity to domain 2 and domain 4, and no binding activity to domain 1. Bispecific antibody 8A7-2A6 displayed similar binding capacity with 8A7, with a slight enhancement of binding value of domain 2 and domain 4. 2A6-8A7 showed binding activity to domain 2, domain 3, and domain 4, but with higher EC_50_ values (EC_50_ = 0.20 nM for domain 3) compared to 8A7, which indicated that the domain specificities of bispecific antibodies were more related to the outer variable region. To elucidate the binding patterns of antibodies to PA, we employed a biolayer interferometry assay (BLI) to examine the binding of antibodies to PA in different sequential orders (Fig. 2C). The results showed that 2A6 or 8A7 does not interfere with the binding to PA of each other, suggesting that their epitopes on PA are likely non-overlapping. The binding of 2A6-8A7 to PA was not affected by other antibodies. However, when 8A7 was pre-bound to PA, the subsequent binding of 8A7-2A6 to PA was eliminated due to competitive effects, indicating that the pre-binding of the 8A7 domain may sterically hinder the interaction between 8A7-2A6 and PA.

The neutralizing activity of bispecific antibody molecules compared with their parental antibodies was further validated at the cellular level and in animal models. The TNA results demonstrated that 2A6-8A7 exhibited the highest neutralizing activity *in vitro* with an IC_50_ of 0.2925 nM, which was superior to the combination of the parental antibodies 2A6 and 8A7 (IC_50_ = 0.9567 nM). On the contrary, the neutralizing activity of 8A7-2A6 (IC_50_ = 5.687 nM) was weaker than that of the combination of the parental antibodies (Fig. 2D). Toxicity experiments in animal models further validated the strong neutralizing activity of 2A6-8A7, which provided 100% protection even at a molar ratio of 0.5:1 to the toxin and significantly prolonged the survival time of rats at a molar ratio of 0.3:1 (Fig. 2E). The mixture of 2A6 and 8A7 could protect rats against toxin at a molar ratio of 0.5:1 to the toxin but failed at a molar ratio of 0.3:1. The 8A7-2A6 exhibited poorer neutralizing activity in animal models as all rats treated with 8A7-2A6 died within 220 min post-challenge even at a molar ratio of 1:1 to toxin. These results revealed that the bispecific antibody could confer a superior effect to the combination of the parental antibodies, and the function of bispecific antibodies depends on their formation.

### The combination of 2A6 and 8A7 promotes PA aggregation and inhibits the PA pore formation

Anthrax lethal toxin is composed of PA and LF. PA can bind to cell surface receptors and facilitate the translocation of LF into the cell. LF, a metalloprotease, specifically cleaves mitogen-activated protein kinase kinases (MKKs) such as MEK1, MEK2, MKK3, MKK4, MKK6, and MKK7. These kinases normally activate specific MAPK signaling pathways and thereby orchestrate cellular responses to a variety of stimuli (24). To accomplish the specific enzymatic cleavage of MKKs, PA and LF undergo the following steps: initial binding of PA83 to cell surface receptors, enzymatic cleavage of PA83, polymerization of PA63, binding of LF to PA63 heptamers, conformational alterations of the polymer, and subsequent internalization of LF into the cell, where it cleaves MKKs. Antibodies could confer neutralizing activity through intervening at any of these sequential stages.

**Fig 3:**
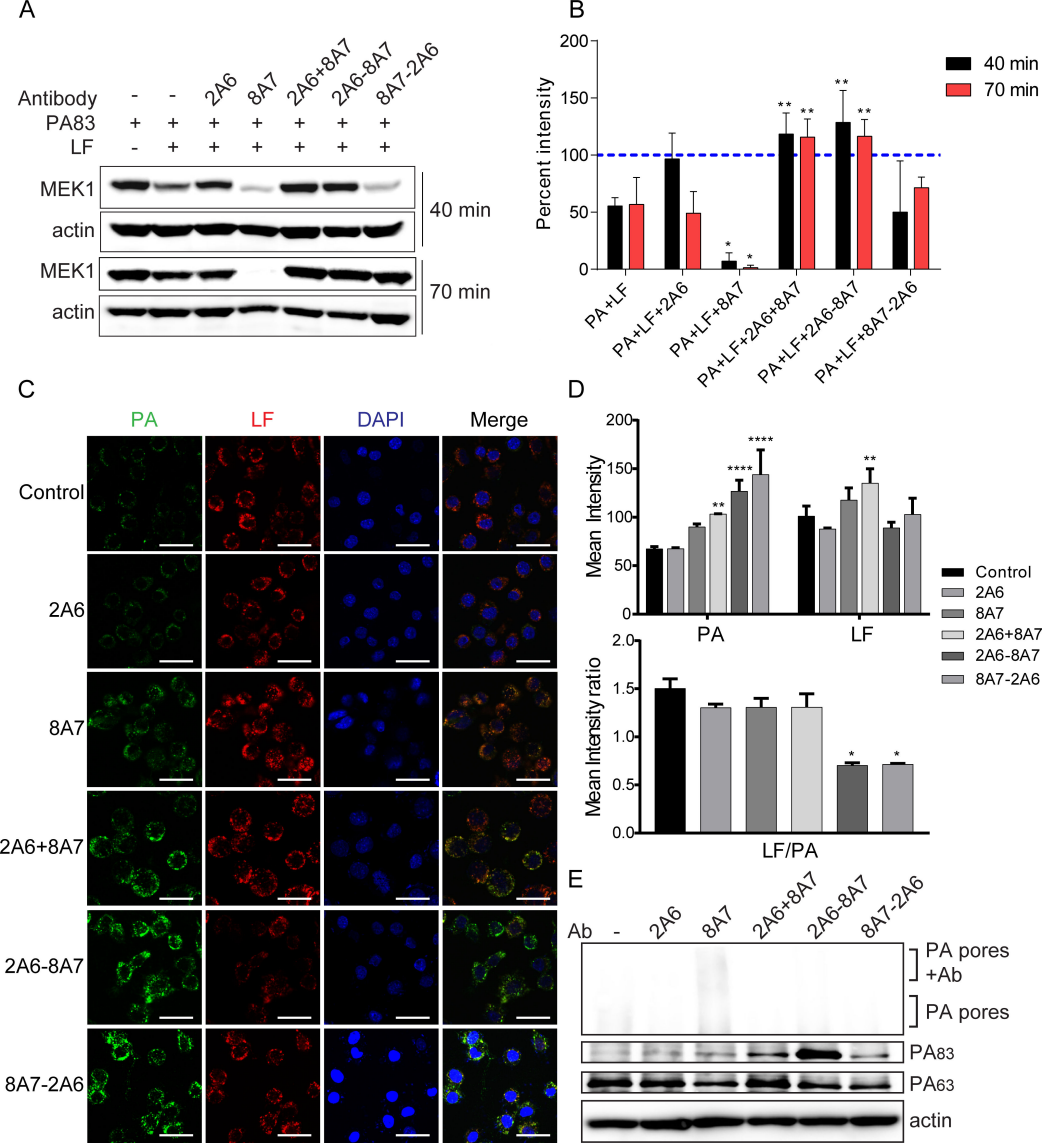
Identification of the neutralizing mechanism of antibodies. (**A**) Western blot verification of antibody inhibition of LF cleavage of MEK1. (**B**) Grayscale analysis on image A and the percent intensity compared with the group without adding LF. Data were analyzed via two-way analysis of variance (ANOVA). Multiple comparisons were performed with group PA+LF as the reference group, *P < 0.05, **P < 0.01. (**C**) PA labeled by FITC and LF labeled by Cy5.5 were added to J774A.1 cell with or without preincubation of antibodies. The cells were imaged by fluorescence microscopy. Scale bar, 30 µm. (**D**) The fluorescence intensity of each group in image C was calculated and compared with the control group. Data were analyzed via two-way ANOVA, **P < 0.01, ****P < 0.0001. The mean intensity ratio of PA/LF in each group was calculated, and data were analyzed via one-way ANOVA, *P < 0.05. (**E**) Western blot analysis of the inhibition of the antibody in PA binding to the cell surface and incorporation into the cell.

To explore the mechanisms by which the antibodies neutralize LeTx, we first examined whether the antibodies could prevent the entry of LF into the cytoplasm, which was indicated by the percentage of the enzymatic cleavage of MEK1 by LF. Compared with the group without incubation of LF, the cells incubated with LF for 40 or 70 min displayed 50% cleavage of MEK1. The addition of 8A7 resulted in nearly 100% cleavage of MEK1, indicating enhanced cleavage of MEK1 by LF (Fig. 3A and B), which was consistent with the improved toxic effect of LeTx by 8A7 (25). The antibody 2A6 could inhibit the cleavage of MEK1 by LF at 40 min after incubation but lost its suppressing activity at 70 min after incubation (Fig. 3A and B). This result might be due to the poor affinity of 2A6 to PA, which could not maintain a persistent bound to PA and thereby failed to inhibit the cleavage of MEK1. The combination of mAb 2A6 and 8A7, as well as the bispecific antibody 2A6-8A7, exhibited significant inhibition of MEK1 cleavage by LF, whereas the bispecific antibody 8A7-2A6 showed only limited inhibition after 70 min of incubation.

To understand the impact of antibodies on the function of PA, we carried out immunofluorescence assays to detect the amounts of PA and LF bound to the J774A.1 cells and western blot experiments to measure the amounts of PA83, PA63, and PA oligomers in target cells after incubation with PA and LF in the presence of the antibodies. Compared with the control, 8A7 significantly induced aggregation of PA and LF around cells, whereas 2A6 showed no such effect. This aggregation induced by 8A7 is consistent with its ability to enhance toxin-mediated cell death, partially explaining its increased toxicity (Fig. 3C and D). Furthermore, in the 8A7 group, we observed an increase in heat- and SDS-stable PA oligomers (known as PA pores) alongside a decrease in PA63. Additionally, we detected a higher molecular weight complex, which we speculate is composed of 8A7 bound to these PA pores (Fig. 3E). We assume that 8A7 promoted the aggregation of PA on the cell surface but also facilitated PA pore formation, which might cause the increase of PA pores and the reduction of the detected PA63. Antibody 2A6 inhibited the formation of PA pores (Fig. 3E), which could partly explain the inhibition of MEK1 cleavage by 2A6.

The combination of 2A6 and 8A7 exhibited both characteristics of the two antibodies. It not only promoted the aggregation of PA and LF compared to the control group, but also inhibited the formation of PA pores. The PA pores aggregated by 8A7 may give benefits for 2A6 to bind PA more stably, which could partly explain the neutralization of this combination against LeTx in the TNA experiments and the enhanced inhibition of MEK1 cleavage.

### 2A6-8A7 introduces a distinct neutralizing mechanism, inhibition of furin enzyme-mediated PA83 cleavage, from the parental mAbs

**Fig 4:**
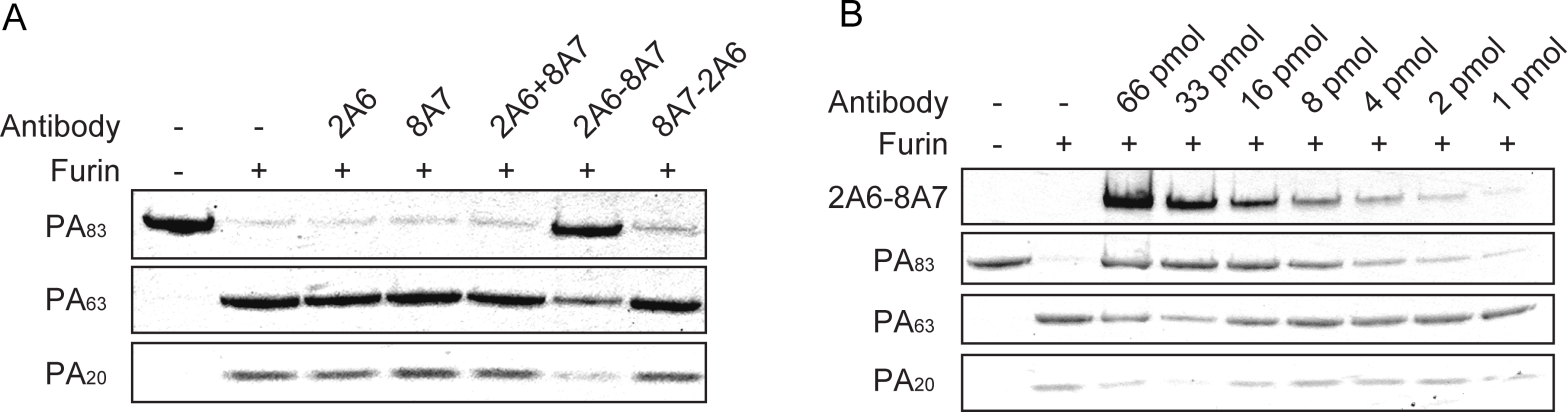
Verification of the introduction of a distinct neutralization mechanism. (**A**) SDS-PAGE validation of the inhibition of antibodies to the cleavage of PA by the furin enzyme. (**B**) The concentration dependence of the inhibitory activity of 2A6-8A7 was verified by SDS-PAGE.

The bispecific antibodies 2A6-8A7 and 8A7-2A6 aggregated even more PA than 8A7, but the amount of involved LF was less than the 8A7 group, resulting in a decreased LF/PA ratio (Fig. 3C and D). This suggests that the bispecific antibody retains the PA-aggregating function of 8A7, while the other parent antibody, 2A6, may contribute through an additional mechanism. The WB results demonstrated that 2A6-8A7 and 8A7-2A6 both inhibited the formation of PA pores, which might partly explain the enhanced neutralization of LeTx *in vitro*. The lack of protective efficacy of 8A7-2A6 in animal models might be the result of the interference of antibody stability or binding affinity from the complicated inflammatory microenvironment and the effect of competitive molecules such as host cell receptors and other serum proteins. Interestingly, compared with the other groups, 2A6-8A7 reduced the enzymatic hydrolysis of PA83 and the production of PA63, suggesting that 2A6-8A7 could inhibit the enzymatic cleavage of PA83 into PA63 upon binding to cell surface receptors (Fig. 3E). Further validation of this hypothesis was performed through the inhibition experiments of furin enzymatic cleavage of PA83. The results showed that 2A6-8A7 inhibited the cleavage of PA83 by furin enzyme in a concentration-dependent manner (Fig. 4A and B), indicating that 2A6-8A7 exhibits a neutralization mechanism distinct from that of its parental antibodies.

### Structural analysis of 2A6 and 8A7 binding to PA

**Fig 5:**
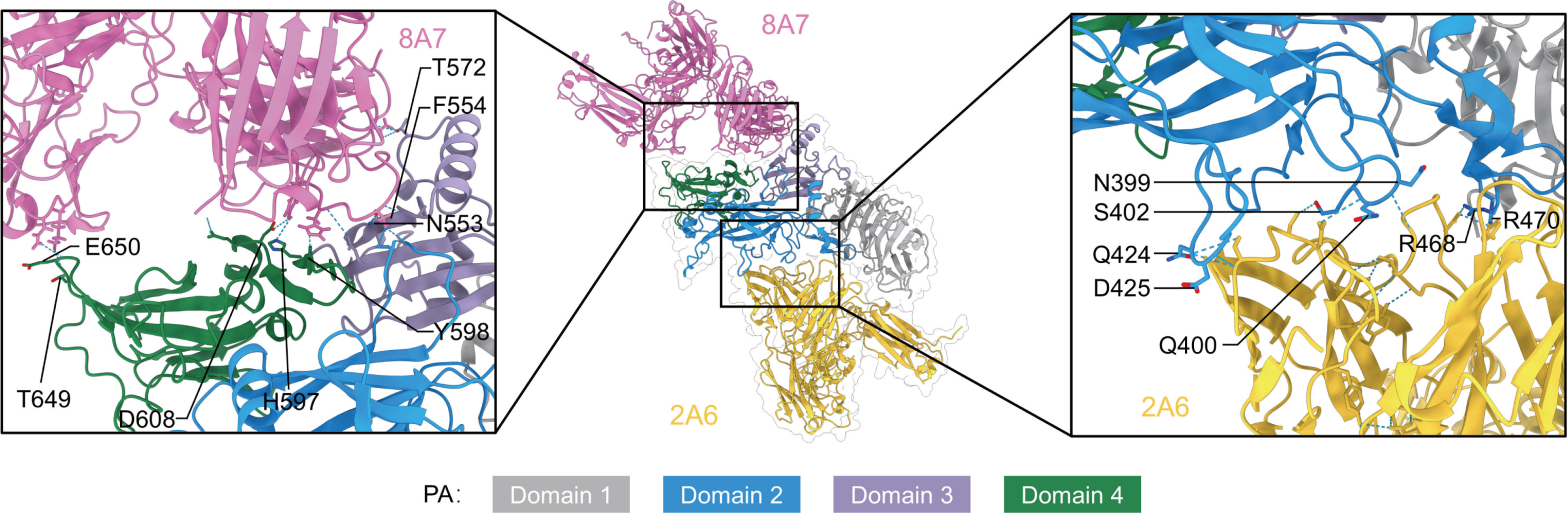
Structures of PA in complex with antibodies. Diagram of PA combined with 8A7 and 2A6, where 8A7 is represented in bright pink, 2A6 in ginger yellow. The 1–4 domains of PA were colored in gray, blue, purple, and green, respectively. The details of the residues on the binding interface of PA to each antibody were displayed.

Structural analysis was performed to explore the epitopes recognized by 2A6 and 8A7. The structures of 2A6 and 8A7 were constructed using AlphaFold2, and their targeted epitopes were predicted using FRODOCK with the structure of PA (PDB: 4H2A). The structural results demonstrated that 8A7 interacted with residue N553, F554, T572, H597, Y598, D608, T649, and E650 of PA (Fig. 5). These residues were located on PA domain 3 (residues 488–595) and domain 4 (residues 596–735), consistent with the strong binding capacity of 8A7 to PA domain 3 and the function of PA oligomerization. The ELISA and SPR results revealed that 8A7 could bind PA with high affinity, but 8A7 could not inhibit the cleavage of MEK1. Instead, 8A7 enhanced the cleavage of MEK1. Combined with the predicted structural information of 8A7 binding with PA domain 3 and domain 4, which mediate self-association of PA63 and cellular receptor binding, respectively, we presume that the binding sites of 8A7 were distinct from the receptor-binding area or the self-association function area of PA and thus 8A7 did not interfere with these functions. PA domain 4 was reported to be involved in the PA-ANTXR2 interface. The predicted structure of 8A7 and PA displayed that the interaction interface between 8A7 and PA is far away from the binding area of PA to ANTXR2, which may explain why 8A7 could not inhibit the binding of PA to the target cell.

The epitope predicted for 2A6 is located at the loop of domain 2, involving residues N399, Q400, S402, Q424, D425, R468, and R470 (Fig. 5). The 2A6 only binds the entire PA molecule with a fast dissociation rate, which may be explained by the flexible loop structure. Domain 2 is associated with the pore formation process, and the structural results may explain why 2A6 is responsible for the inhibition of PA pore formation. Based on the structural results, 2A6 and 8A7 could simultaneously bind to PA on distinct epitopes and thus could retain both functions of the two mAbs when used in combination. 2A6-8A7 may crosslink different PAs and interfere with the cleavage process through steric hindrance and therefore introduce distinct inhibition mechanisms from the parental mAbs.

## DISCUSSION

Here, we report the functions and mechanisms of the antibody strategies against anthrax PA that combine two non-protective antibodies against different epitopes on the PA protein as a promising way to improve the efficacy of antibody therapeutics for anthrax. We show that a certain combination of 2A6 and 8A7 could exert neutralizing activity against anthrax LeTx. Furthermore, the DVD-Ig bispecific antibody derived from the two mAbs potently neutralized LeTx in cellular assays and animal models and revealed a unique neutralizing mechanism. We show that non-blocking, non-neutralizing epitopes could provide unexpected benefits and boost the potency of antibodies when combined or in a bispecific format. Thus, combining antibodies targeting non-neutralizing epitopes can be a useful method to regain better efficacy of previously less active antibodies and enable the generation of more potent neutralizing antibodies.

**Fig 6:**
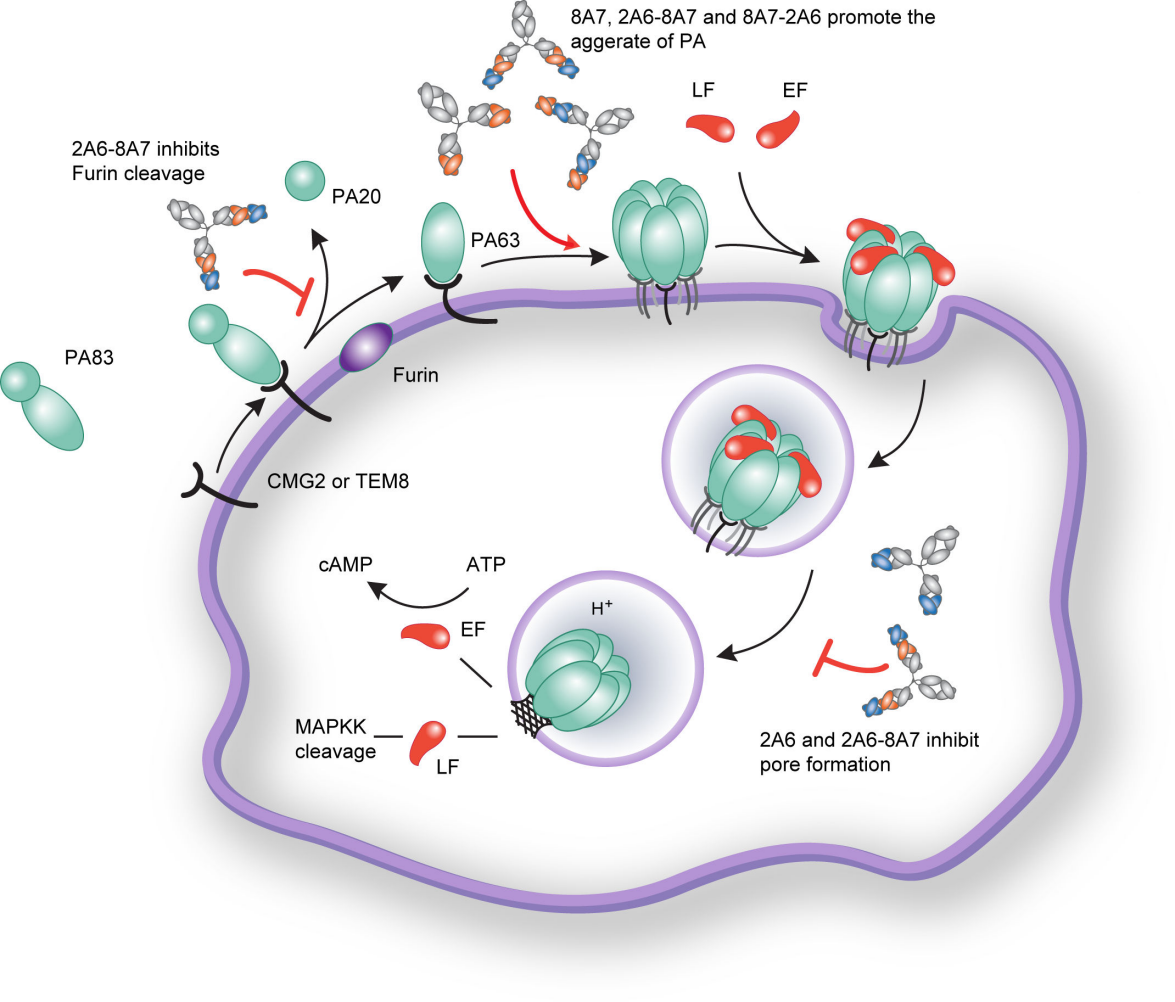
Neutralizing mechanisms of antibodies. The role and mechanism of antibodies in the process of PA functions. Furin cleavage was inhibited by 2A6-8A7. The aggregation of PA was induced by 8A7, 2A6-8A7, and 8A7-2A6. The conformational changes of pre-pore proteins were inhibited by 2A6 and 2A6-8A7, thus preventing the release of LF and the cleavage process of MEK1.

The antitoxin strategy reported here, which combines two non-protective antibodies, is a novel approach to combat anthrax exposures. So far, the antibodies targeting PA have been reported to confer neutralizing activities through several mechanisms: blocking PA binding to cell receptors such as IQNPA, ETI-204, and Abthrax (Raxibacumab) (8, 26, 27), inhibiting PA cleavage by furin such as 48.3 and Mab 7.5G (10, 28), inhibiting the PA heptamerization such as MAb1303 (29), inhibiting the conversion of the PA oligomer from "pre-pore" to "pore" conformation such as JKH-C7 (30, 31), and inhibiting the endocytosis and translocation of anthrax toxin such as cAb29 (32, 33). Despite the lack of protective activity of 8A7 and 2A6, the combination of 2A6 and 8A7, as well as the engineered bispecific antibody 2A6-8A7, could achieve full protection against LeTx challenge. Regarding the neutralization mechanism of the 2A6 and 8A7 combination, we speculate that the crosslinking of 8A7 and PA favors 2A6 to bind the 8A7-PA complex more efficiently, thus exerting a robust inhibition of the PA pore formation and the release of LF (Fig. 6). As for the bispecific antibody 2A6-8A7, a possible explanation for its neutralizing capacity is that 2A6-8A7 could interfere with different stages during the function of the LeTx (Fig. 6). First, 2A6-8A7 effectively inhibits the enzymatic cleavage process of PA83 by the furin enzyme upon its binding to cell surface receptors, such as CMG2 or TEM8 (2). Second, 2A6-8A7 promotes the aggregation of PA on the J774A.1 cell surface but not LF. Third, as the polymer of PA and LF enters the cell, the conformational change of PA from pre-pore to pore structure, which enables the release of LF into the cytosol, is blocked by 2A6-8A7. The engineering of bispecific antibodies is not only a simple addition of the functions of their parental mAbs, but also can introduce new neutralization mechanisms. The 2A6-8A7 not only retains the functions of 2A6 to inhibit the formation of PA pores, as well as the function of 8A7 to promote the aggregation of PA. Besides, it also introduces a new mechanism for inhibiting PA83 cleavage.

The improved efficacy conferred by the combination of non-protective antibodies was also reported in other diseases. The binding of non-neutralizing antibody MR235 to Marburg virus glycoprotein increased the accessibility of epitopes in the receptor-binding site for neutralizing mAbs, resulting in enhanced virus neutralization by other mAbs (19). The non-neutralizing antibody FVM09, targeting Ebola virus glycoprotein, has almost no neutralizing activity by itself. However, its combination with poorly neutralizing antibody m8C4 showed 70-fold improvement in neutralizing efficacy (18). This phenomenon may be related to the antigenic conformational changes upon the first antibody binding that lead to better access of the second antibody to its epitope. In another study about anthrax toxin-enhancing mAb N1F7 and protective mAb N2D6, in the presence of a constant amount of N1F7, the N2D6 exhibited about 100-fold improved neutralizing capacity (17). These findings suggest that we could pay more attention to those non-protective mAbs and toxin-enhancing mAbs, which may exert neutralizing effects when combined with other molecules.

Overall, this study explored the strategies using two non-protective antibodies against anthrax, which could serve as candidate drugs to protect against anthrax infections. These findings provide a reference for subsequent infectious disease-related treatment methods and a new approach for the research and application of non-protective antibodies. The study of the mechanism of bispecific antibodies provides effective information for studying *Bacillus anthracis* infection and immune processes.

## MATERIALS AND METHODS

### Antibody sequences

The sequences of antibodies 2A6 and 8A7 were obtained in our previous study (25). The variable regions of 2A6 and 8A7 were connected through the natural flexible near C region linker (the heavy chain linker: ASTKGP, DNA sequence GCCTCCAACAAGAGGGCCCA; the light chain linker: TVAAP, DNA sequence ACTGTGGCTGCCCA). The dual variable regions were then connected to the constant regions of the heavy and light chains of the antibody to construct a full-length molecule with dual variable regions. 2A6 and 8A7 were, respectively, placed at the N-terminus, and subsequently, the 2A6-8A7 DVD-Ig and 8A7-2A6 DVD-Ig were constructed. The sequences of the bispecific antibodies were cloned into the expression vector pcDNA3.4. Sequences are given in Table 1.

**TABLE 1:** Protein sequences of antibodies

No.	Antibody	Sequence
1	Ig heavy chain variable region, antibody 2A6	QVQLQESGPGLVKPSQTLSLTCTVSGGSISSGGYYWSWIRQHPGKGL EWIGYIYYSGRTYYNPSLKSRVTMAVHTSKNQYSLRLSSVTAADTAV YFCATGDGGVAGTFDFWGQGNLVTVSS
2	Ig light chain variable region, antibody 2A6	EIVMTQSPASLSVSPGEGATLSCRASQSIHSNLDWYQQKPGQAPRLLI YGASTRATGVPARFSGSVSGTEFTLTISSLQSEDFAVYYCQQYNNWPL TFGGGTKVEIKR
3	Ig heavy chain variable region, antibody 8A7	EVQLLESGGGLVQPGGSLRLSCAASGFSFSSYAMSWVRQAPGTGLE WVSGISGSGDNIYYADSVKGRFTISRDNSKNTVYMQMNSLRAEDTAL YYCAKFSGHYGSGSFHLPEYFQHWGQGTLVTVSS
4	Ig light chain variable region, antibody 8A7	DIQMTQSPATLSASIGDRVTITCRASQSISSWLAWYQQKPGKAPKVLI YKASRLESGVPSRFSGSGSGTEFTLTISSLQPDDFATYYCQQYNTYWT FGQGTKVEIIR
5	Ig heavy chain constant region	ASTKGPSVFPLAPSSKSTSGGTAALGCLVKDYFPEPVTVSWNSGALTS GVHTFPAVLQSSGLYSLSSVVTVPSSSLGTQTYICNVNHKPSNTKVDK KVEPKSCDKTHTCPPCPAPELLGGPSVFLFPPKPKDTLMISRTPEVTC VVVDVSHEDPEVKFNWYVDGVEVHNAKTKPREEQYNSTYRVVSVLT VLHQDWLNGKEYKCKVSNKALPAPIEKTISKAKGQPREPQVYTLPP SRDELTKNQVSLTCLVKGFYPSDIAVEWESNGQPENNYKTTPPVLDSD GSFFLYSKLTVDKSRWQQGNVFSCSVMHEALHNHYTQKSLSLSPGK
6	Ig light chain constant region	TVAAPSVFIFPPSDEQLKSGTASVVCLLNNFYPREAKVQWKVDNALQ SGNSQESVTEQDSKDSTYSLSSTLTLSKADYEKHKVYACEVTHQGLSS PVTKSFNRGEC
7	Ig heavy chain natural flexible linker	ASTKGP
8	Ig light chain natural flexible linker	TVAAP

### Expression and purification of bispecific antibodies

The bispecific antibodies were expressed in Expi293F cells by transfecting the plasmids with ExpiFectamine 293 transfection kit (Gibco) at 37°C under 5% CO_2_ in a cell culture shaker (Infors HT) at 120 rpm according to the manufacturer’s protocol. Supernatants were collected by centrifugation at 4°C and 8,000 rpm after a 144 h incubation. The antibodies were captured by Protein A affinity chromatography column, washed with PBS (pH 7.4), eluted with 0.1 M glycine (pH 2.7), and then immediately balanced by 1 M Tris-HCl (pH 9.0). The purified antibodies were exchanged in PBS (pH 7.4), and the concentrations were then measured using the BCA Protein assay kit and stored at −80°C for future use.

### Surface plasmon resonance assay

Antibody-antigen binding kinetics were determined by SPR technology using a Biacore T200 instrument (GE Healthcare). The surface of the CM5 chip was activated by the mix of 0.4 M of EDC (N-ethyl-N′-(3-diethylaminopropyl)-carbodiimide) and 0.1 M of NHS (N-hydroxysuccinide) in a volume ratio of 1:1 at a flow rate of 10 µL/min for 7 min. The anti-human Fc antibody was diluted in sodium acetate (10 mM, pH 5.0) to a concentration of 20 µg/mL and then subjected to the CM5 chip at a flow rate of 10 µL/min for 5 min, and the unbound site was blocked with ethanolamine (1 M, pH 8.5) at a flow rate of 10 µL/min. The tested antibody was captured onto channel 2. The PA protein was diluted into a serial concentration and tested through channels 1 and 2 using the single-cycle kinetics method at a flow rate of 30 µL/min. The flow durations were 60 s for the association stage and 180 s for dissociation.

### Enzyme-linked immunosorbent assay

ELISA microplates (Corning) were coated with the four domain proteins of PA or full-length PA at a concentration of 2 µg/mL in 0.05 M carbonate buffer solution (pH 9.6) overnight at 4°C. After three washes with PBST, wells were blocked by 2% BSA in PBST at 37°C for 1 h. After three washes with PBST, antibodies were added to the plates and incubated at 37°C for 1 h. Antibodies were assayed at a 10 nM starting concentration and at seven additional double serial dilutions. After extensive washes, wells were incubated with 1:10,000 diluted HRP-conjugated anti-human IgG secondary antibody (Abcam) for 1 h at 37°C. Finally, the TMB single-component substrate solution (Solarbio) was added to the plates and incubated at room temperature for 6 min, and 2 M H_2_SO_4_ was then added to stop the reaction. The absorbance at 450 and 630 nm was measured using a microplate reader (Spectra Max 190, Molecular Devices).

### Biolayer interferometry assay

The PA protein was biotinylated with EZ-Link NHS-Biotin (ThermoFisher) according to the manufacturer’s instructions. The PA-biotin was then immobilized onto SA biosensors (Gator), followed by a 60 s washing step. The biosensors were then immersed in wells containing antibodies 1 and antibodies 2 for 180 s, sequentially. The results were monitored by Gator Prime and then analyzed using GraphPad Prism 5.

### Toxin neutralization assay

J774A.1 cells were inoculated into a 96-well plate and incubated overnight at 37°C in a 5% CO_2_ environment. PA and LF were diluted to 100 ng/mL in MEM + 10% FBS medium and incubated with serially diluted test antibodies at 37°C for 1 h. The mAbs combination of 2A6 and 8A7 was premixed at different ratios and then incubated together with PA and LF. Death control (toxin only) and survival control (medium only) were set up. The toxin-antibody mixtures were added to the cell wells and allowed to incubate for 4 h. The supernatant was then replaced with 1 mg/mL MTT (3-(4,5-dimethylthiazol-2-yl)-2,5-diphenyltetrazolium bromide) and allowed to incubate for another 4 h. Then, the cell culture supernatant was discarded, and the extraction buffer was added. The absorbance was measured at 570 nm, with 630 nm as the reference wavelength. Cell viability was calculated as (OD_measurement_ − OD_death control_)/(OD_survival control_ − OD_death control_). The antibody concentration versus cell survival curve was fitted using GraphPad Prism 5 to determine the IC_50_ of the mAb.

### *In vivo* neutralization experiment

The Fisher 344 rats (Vital River Laboratory Animal Technology Co. Ltd.), weighing 160–180 g, were randomly divided into groups (five animals each group) and challenged with 10 µg PA and 10 µg LF per animal. The tested antibodies were premixed with the toxin in a specific molar ratio, and a death control group was set up with only the toxin. The toxin mixture was diluted with PBS to 200 µL and injected through the tail vein. The survival of the rats was observed for 48 h, and the time of death was recorded. The survival curves were plotted using GraphPad Prism 5. All experiments were done in compliance with institutional guidelines and have been approved by the Institutional Animal Care and Use Committee of the Laboratory Animal Center of the Academy of Military Medical Sciences.

### Western blot

To detect the inhibition of the MEK1 cleavage by the antibodies, J774A.1 cells were inoculated into a 6-well plate and incubated overnight at 37°C. A mixture of the tested antibody and PA83, and LF in the molar ratio of 1:1:1 was incubated at 37°C for 1 h before being added to the cell wells. Cells were incubated with the mixture at 37°C for 40 or 70 min. To detect the inhibition of the PA binding by the antibodies, J774A.1 cells were inoculated into a 6-well plate and incubated overnight at 37°C. A mixture of the tested antibody and PA83 in a molar ratio of 1:1 was incubated at 37°C for 1 h before being added to the cell wells and incubated at 37°C for 45 min.

Cells were washed with PBS after incubation and then lysed with RIPA lysate containing protease inhibitor. Cell lysates were clarified by centrifugation at 12,000 rpm at 4°C for 10 min, mixed with 1:5 (vol/vol) 6× SDS-loading buffer. After SDS-PAGE and transferred to nitrocellulose membrane, the membranes were then blocked with skim milk at room temperature for 1 h. Primary antibodies against MEK1 (Abcam) or PA (laboratory reserved) were added at a 1:200 or 1:500 dilution in skim milk and incubated on a shaker at 4°C overnight. After three washes in TBST, the membranes were incubated with HRP-conjugated anti-rabbit IgG secondary antibody (Abcam) at a 1:5,000 dilution for 1 h at 37°C. The membranes were washed with TBST and incubated with Western HRP Substrate (Millipore) at room temperature. The immunoreaction was detected using the chemiluminescence system. The membranes were then soaked in stripping solution for 10 min. After three washes in TBST and blocking in skim milk, the membranes were hybridized with HRP-conjugated anti-actin antibody (Abcam) at 37°C for 1 h at a 1:5,000 dilution. The membranes were then washed three times in TBST and incubated with Western HRP Substrate at room temperature. The immunoreaction was detected using the chemiluminescence system. The images were analyzed using Image J.

### Immunofluorescence

J774A.1 cells were inoculated on the glass slide in a 12-well plate and incubated overnight at 37°C. PA labeled by FITC and LF labeled by Cy5.5 were diluted with culture medium and incubated with the tested antibody for 30 min. Subsequently, the mixture was added to the cell wells for an incubation of 30 min. The cells on the glass slide were rinsed twice with PBS and then fixed with 4% paraformaldehyde at room temperature for 10 min, followed by washing twice with PBS for 5 min. Afterward, the cells were blocked with a blocking solution (PBS containing 2% BSA) at room temperature for 30 min. The glass slide was then flipped onto the slide, sealed with DAPI sealant, and incubated at room temperature for 30 min. Finally, the slide was observed under a fluorescence microscope.

### SDS-PAGE

To verify the purity of the antibodies, samples were mixed with reducing or non-reducing SDS-loading buffer, respectively, and separated through SDS-PAGE at a 150 V voltage. To verify the inhibition of antibodies in furin enzyme cleavage of PA and PA63 heptamer formation, 66 nmol and serially diluted antibodies were mixed with 36 nmol PA83, and a control group without the tested antibody was set up. The furin enzyme cleavage reaction system was prepared in a final volume of 20 µL using 1M Tris to ensure that PA63 remains a monomer after furin enzyme cleavage. Furin enzyme (1 unit) at the volume of 0.5 µL was added into each tube after an incubation at 37°C for 1 h, then incubated with the system at 37°C for 1 h. The heptamer reaction system was prepared using 50 mM MES (pH 5.5) with a final volume of 20 µL. The samples were mixed with 1:5 (vol/vol) 6× SDS-loading buffer (non-reducing) and separated through SDS-PAGE.

### Structure and epitope prediction of PA for antibodies

The light and heavy chain sequences of the antibodies were uploaded together to AlphaFold2 for homologous modeling, resulting in the modeling structure files of the antibody. Subsequently, the PA structure (PDB_ID: 4h2a) and the antibody modeling structure were submitted to the WeMol cloud platform (https://wemol.wecomput.com/) for molecular docking using the FRODOCK module. Utilizing ChimeraX 1.8rc software, the hydrogen bonds between amino acid residues at the interface were indicated and annotated.

### Statistical analysis

For the TNA and ELISA assays, the EC_50_ values for antibodies were determined using four-parameter nonlinear regression (GraphPad Prism 5). For mouse survival curves, data were analyzed via the Log-rank (Mantel-Cox) test. For the SPR assay, affinity values, including association rates (*K*_on_), dissociation rates (*K*_off_), and affinity constants (*K*_*D*_), were calculated using Biacore T200 Evaluation Software with a 1:1 binding model. For western blot and immunofluorescence, the statistical analyses were conducted using GraphPad Prism 5 using unpaired two-tailed Student’s *t*-tests unless otherwise specified. The level of significance was defined at *P < 0.05, **P < 0.01, ***P < 0.001, and ****P < 0.0001.

## Data Availability

The sequences of the antibodies can be found in Table 1.
